# Retraction Note: Magnetic triazine-based dendrimer as a versatile nanocarrier for efficient antiviral drugs delivery

**DOI:** 10.1038/s41598-024-72209-1

**Published:** 2024-09-16

**Authors:** Rezvan Ahangarani-Farahani, Mohammad Ali Bodaghifard, Sajad Asadbegi

**Affiliations:** 1 Department of Chemistry, Faculty of Science, Arak University, Arak, 38156-88349 Iran; 2 Institute of Nanosciences and Nanotechnology, Arak University, Arak, 38156-88349 Iran

Retraction of: *Scientific Reports* 10.1038/s41598-022-24008-9, published online 14 November 2022

The Editors have retracted this Article. After publication of this Article, concerns were raised about the Fourier-transform infrared spectroscopy (FT-IR) data presented in Figure 2 and the X-ray diffraction (XRD) data presented in Figures 3c and 8a. Specifically,

- In Figure 2, the FT-IR spectra show several data points where two different values of transmittance correspond to a single wavenumber. This is not physically possible for this type of experiment;
- In Figures 3c and 8a, two XRD spectra are presented for Fe3O4@SiO2@TAD-G3. These spectra do not appear to correspond to the same material.

The Authors provided raw data on request from the editors. However, this was insufficient to satisfy the concerns raised. The Editors therefore no longer have confidence in the results and conclusions presented.

Mohammad Ali Bodaghifard disagrees with this retraction. Rezvan Ahangarani-Farahani and Sajad Asadbegi did not respond to our correspondence about this retraction.

